# Supplementary material for: Immunoproteasome Inhibition Impairs Differentiation but Not Survival of T Helper 17 Cells
Source: Cells. 2025 May 10;14(10):689. doi: 10.3390/cells14100689 (PMC12109796; doi:10.3390/cells14100689)
Supplement: Supplementary file 1 [file cells-14-00689-s001.zip › cells-3578116-supplementary.pdf]

Supplementary Information

Immunoproteasome inhibition impairs differentiation but not survival of T helper 17 cells

Franziska Oliveri[1,2,†], Dennis Mink[1,2], Tony Muchamuel[3], and Michael Basler[1,2,*]

1 Biotechnology Institute Thurgau at the University of Konstanz, 8280 Kreuzlingen, Switzerland
2 Division of Immunology, Department of Biology, University of Konstanz, 78457 Konstanz, Germany
3 Department of Research, Kezar Life Sciences, South San Francisco, CA 94080, USA
* Correspondence: michael.basler@bitg.ch

† Current address: Department of Otorhinolaryngology, Head and Neck Surgery, Ulm University Medical Center, 89075 Ulm, Germany

Supplementary Table S1: List of antibodies.

Antigen	Fluorochrome	Clone	Dilution	Supplier
CD3	APC	145-2C11	1:400	Biolegend
CD3	eFluor450	145-2C11	1:400	eBioscience
CD4	BV605	GK1.5	1:1200	Biolegend
CD4	PE	GK1.5	1:1600	Biolegend
CD4	APC	GK1.5	1:1600	Biolegend
CD11c	BV421	N418	1:200	Biolegend
CD44	PE-Cy7	IM7	1:1600	eBioscience
CD62L	BV421	MEL-14	1:800	Biolegend
CD223	PE	C9B7W	1:200	Biolegend
PD-1	PE	29F.1A12	1:200	Biolegend
CD3	unlabeled	145-2C11	1:100	Biolegend
CD28	unlabeled	37.51	1:100	Biolegend
IL-17A	APC	ebio17B7	1:800	eBiosciences
FoxP3	eFluor450	FJK-16s	1:200	eBioscience

Supplementary Figure S1

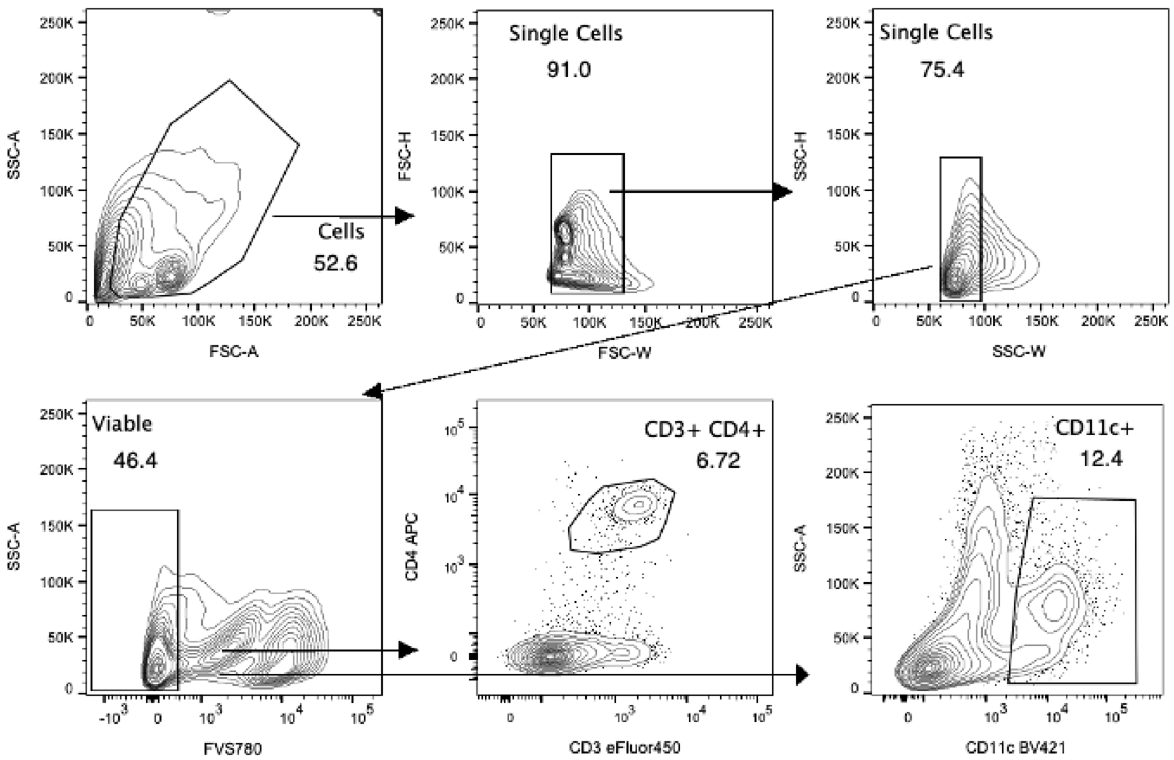

Gating scheme for the identification of CD4$^+$ T cells and dendritic cells in colitis experiments. Single cell suspensions from various organs were analyzed by flow cytometry. After the exclusion of debris, doublets and dead cells, T helper cells were identified as CD3$^+$ CD4$^+$ and dendritic cells as CD11c$^+$. An example of the lamina propria is shown here.

Supplementary Figure S2

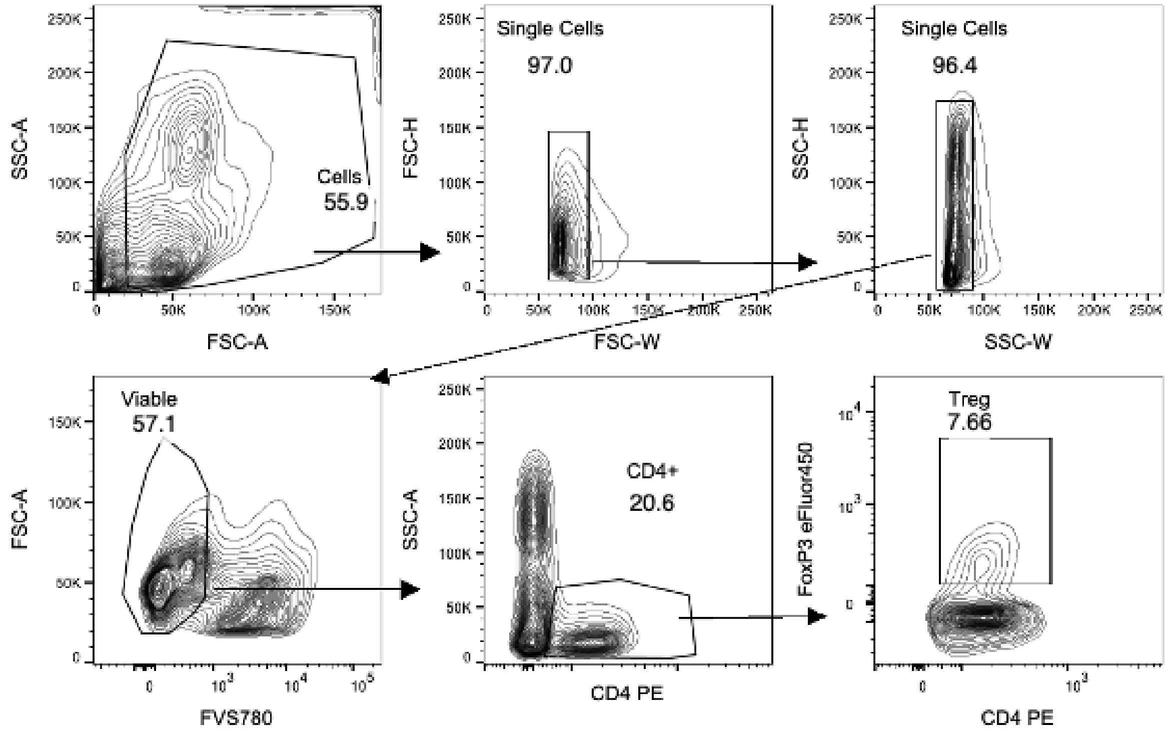

Gating scheme for the identification of CD4$^+$ T cells in HDM-AI experiments. Single cell suspensions from lung and spleen were analyzed by flow cytometry after re-stimulation *in vitro* with PMA/Ionomycin in the presence of Brefeldin A for 5 hours. Intracellular staining was performed after fixation with 4% paraformaldehyde. After the exclusion of debris, doublets and dead cells, CD4$^+$ T cells were identified as SCC-A^{lo} CD4$^+$. An example of the lung is shown here. Subsequently, cells were identified as FoxP3$^+$ Tregs which is shown in the bottom right plot. The same gating, till the identification of CD4$^+$ T cells, was applied for the analysis of Th17 cells. The gating of IL-17A$^+$ Th17 cells for the different treatment groups is shown in main Figure 3.

Supplementary Figure S3

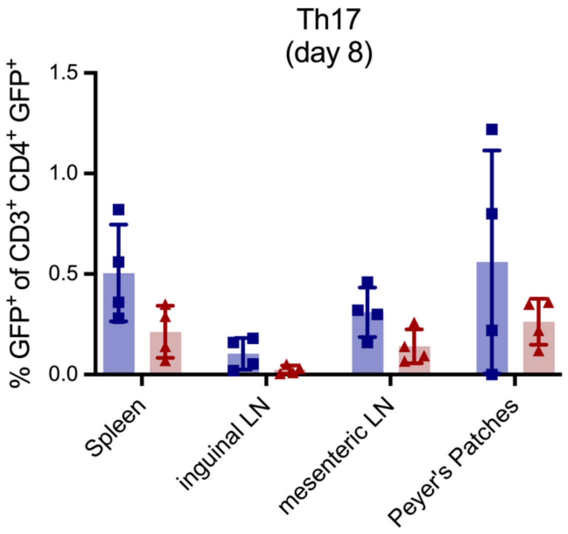

Th17 cells in various organs in DSS-induced colitis. 2 % DSS was added to the drinking water of IL-17A-GFP mice for 5 days, followed by normal drinking water. Simultaneously, vehicle or ONX 0914 (10 mg/kg) was administered subcutaneously every other day and mice were sacrificed on day 8. Flow cytometric analysis was performed on dissected and mechanically/enzymatically dissociated tissues. Frequency of Th17 cells among $CD4^+$ T cells is shown in different organs. Graphs show pooled data of 2 independent experiments, *n*=4. Data is shown as mean ± SD. Each point represents an individual mouse. No statistically significant differences were detected.

Supplementary Figure S4

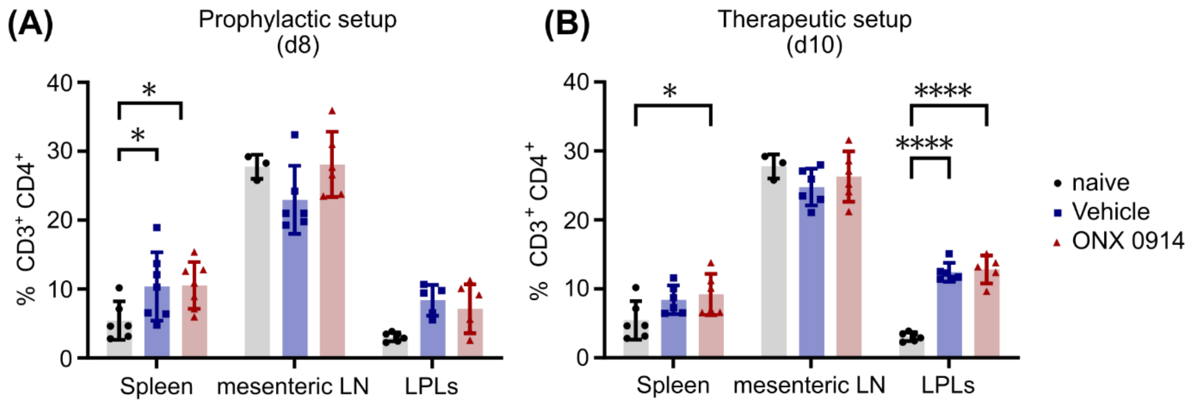

Immunoproteasome inhibition does not change the frequency of bulk CD4$^+$ T cells. 2 % DSS was added to the drinking water of IL-17A-GFP mice for 5 days. **(A)** Simultaneously, vehicle or ONX 0914 (10 mg/kg) was administered subcutaneously every other day and mice were sacrificed on day 8. **(B)** Starting on day 6, vehicle or ONX 0914 (10 mg/kg) was administered subcutaneously every other day and mice were sacrificed on day 10.
(A, B) Frequency of CD3$^+$ CD4$^+$ T helper cells in the spleen, mesenteric lymph node (LN) and among lamina propria lymphocytes (LPLs). Graphs show pooled data of 2-3 independent experiments. *n*=5-7. Data is shown as mean ± SD. Each point represents an individual mouse. * $p < 0.05$, ** $p < 0.01$, **** $p < 0.0001$ (2-way ANOVA).

Supplementary Figure S5

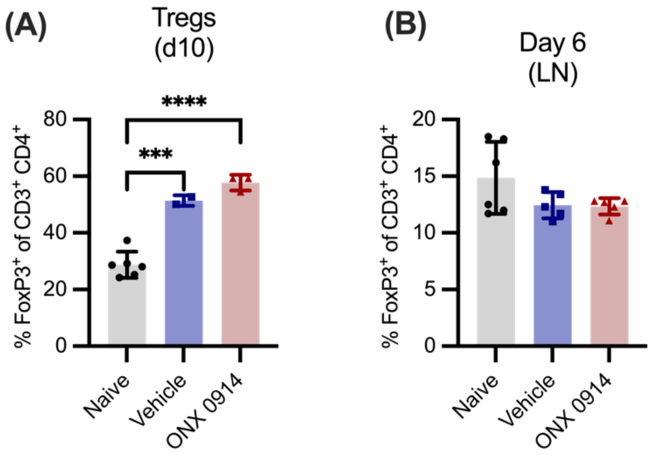

Immunoproteasome inhibition does not affect survival of Tregs in the lamina propria or change their frequency in the mesenteric lymph node. 2 % DSS was added to the drinking water of FoxP3-GFP mice for 5 days. **(A)** Vehicle or ONX 0914 (10 mg/kg) was administered subcutaneously on day 6 and 8 and mice were sacrificed on day 10. Frequency of Tregs cells ($CD3^+$ $CD4^+$ $FoxP3^+$) was analyzed in the lamina propria on day 10 **(B)** Vehicle or ONX 0914 (10 mg/kg) was administered subcutaneously on day 0, 2 and 4 and mice were sacrificed on day 6. Frequency of Tregs ($CD3^+$ $CD4^+$ $FoxP3^+$) in the mesenteric lymph node (LN) was analyzed. *n*=3-6. Data is shown as mean ± SD. Each point represents an individual mouse. *** $p < 0.001$, **** $p < 0.0001$ (1-way ANOVA).

Supplementary Figure S6

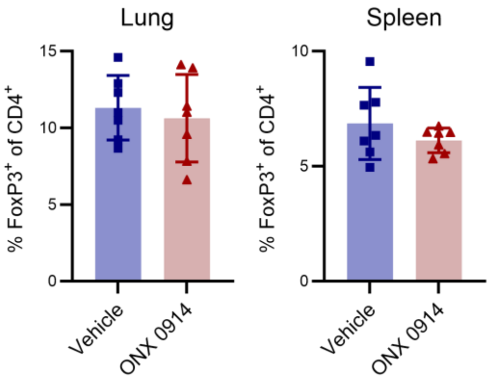

Tregs are not affected by immunoproteasome inhibition in house dust mite-induced airway inflammation (HDM-AI). GATIR mice received four intranasal applications of 50 µg HDM extract on day 0, 7, 14 and 21. One hour before the last immunization, mice were treated with 10 mg/kg ONX 0914 or vehicle subcutaneously. Tregs in the lung (left) and spleen (right) were analyzed on day 23 (gating scheme in supplementary figure S3). Each point represents an individual mouse. Data is pooled from two independent experiments (*n*=7). No statistically significant differences were detected.

Supplementary Figure S7

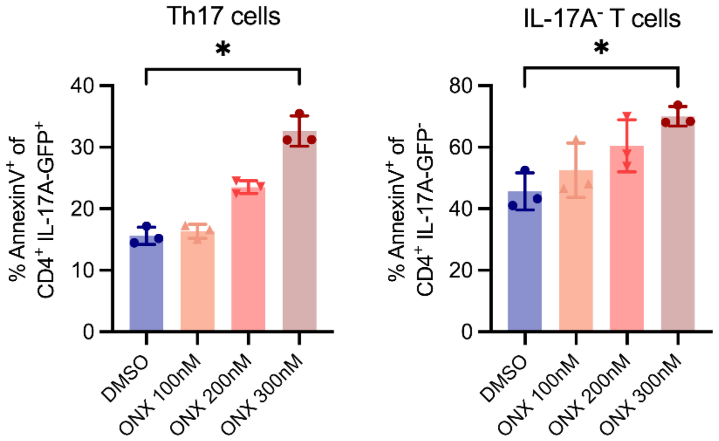

Mild induction of apoptosis by immunoproteasome inhibition in vitro. $CD4^+$ T cells were magnetically isolated from spleens of naïve mice and differentiated into Th17 cells *in vitro*. After 5 days, samples were treated with indicated concentrations of ONX 0914 or vehicle (DMSO) as control. Apoptosis was measured by AnnexinV staining after 24 hours by flow cytometry on IL-17A-GFP^+ $CD4^+$ Th17 cells (left) and IL-17A-GFP^- $CD4^+$ T cells (right) (n=3). Data is shown as mean ± SD, each point per group represents an individual mouse. * $p < 0.05$ (Kruskal-Wallis test or 1-way ANOVA).
